# Age and Gender Effects in Sensitivity to Social Rewards in Adolescents and Young Adults

**DOI:** 10.3389/fnbeh.2019.00171

**Published:** 2019-07-29

**Authors:** Sibel Altikulaç, Marieke G. N. Bos, Lucy Foulkes, Eveline A. Crone, Jorien van Hoorn

**Affiliations:** ^1^Department of Developmental and Educational Psychology, Faculty of Social and Behavioural Sciences, Institute of Psychology, Leiden University, Leiden, Netherlands; ^2^Leiden Institute for Brain and Cognition, Leiden, Netherlands; ^3^Department of Clinical, Neuro and Developmental Psychology, Faculty of Behavioural and Movement Sciences, Vrije Universiteit Amsterdam, Amsterdam, Netherlands; ^4^Department of Education, University of York, York, United Kingdom

**Keywords:** social reward, social context, age, gender, adolescence, SRQ-A

## Abstract

Adolescence is a sensitive period for socio-cultural processing and a vast literature has established that adolescents are exceptionally attuned to the social context. Theoretical accounts posit that the social reward of social interactions plays a large role in adolescent sensitivity to the social context. Yet, to date it is unclear how sensitivity to social reward develops across adolescence and young adulthood and whether there are gender differences. The present cross-sectional study (*N* = 271 participants, age 11–28 years) examined age and gender effects in self-reported sensitivity to different types of social rewards. In order to achieve this aim, the Dutch Social Reward Questionnaire for Adolescents was validated. Findings revealed that each type of social reward was characterized by distinct age and gender effects. Feeling rewarded by gaining positive attention from others showed a peak in late adolescence, while enjoying positive reciprocal relationships with others showed a linear increase with age. Enjoying cruel behavior toward others decreased with age for girls, while boys showed no changes with age and reported higher levels across ages. Reward from giving others control showed a mid-adolescent dip, while enjoying group interactions did not show any changes with age. Taken together, the results imply that the social reward of social interactions is a nuanced and complex construct, which encompasses multiple components that show unique effects with age and gender. These findings enable us to gain further traction on the ubiquitous effects of the social context on decision-making in adolescent’s lives.

## Introduction

Adolescence is the period between childhood and adulthood often characterized by heightened sensitivity to rewards, especially in a social context (Crone and Dahl, 2012; Blakemore and Mills, 2014; van Hoorn et al., 2019). Indeed, studies of non-social rewards in adolescence show greater reward sensitivity in risk-taking tasks involving immediate reward (Weigard et al., 2014), greater sensation seeking in self-report questionnaires (Martin et al., 2002; Steinberg et al., 2017), and more approach behavior toward rewards (Urošević et al., 2012). In the social domain, adolescents are exceptionally attuned to social rejection (Sebastian et al., 2010), quickly embarrassed when observed by peers (Somerville et al., 2013), and susceptible to peer influence (e.g., Chein et al., 2011). Theoretical accounts postulate that adolescents may be highly attuned to the social context because they are more sensitive to *social rewards* (for a review, see Foulkes and Blakemore, 2016). Social reward can be defined as “the motivational and pleasurable aspects of interactions with other people” (Foulkes et al., 2014a, p. 1). Yet to date, the development of sensitivity to social rewards across adolescence and into adulthood is unclear. In addition, few studies have examined the effect of different *types* of social reward across adolescence (Foulkes et al., 2017). The current study aimed to fill this gap by examining age and gender effects in self-reported sensitivity to a range of social rewards in a cross-sectional design including adolescence to young adulthood (ages 11–28 years).

The social world of adolescence encompasses many challenges, and fitting in with the peer group is a key developmental task. During this time, both the quality and the quantity of time spent with peers increases (Somerville, 2013; Lam et al., 2014). Previous work shows that social interactions with peers are experienced as more rewarding for adolescents relative to adults. For example, adolescents feel more rewarded when talking to their peers compared to talking with adults (Csikszentmihalyi et al., 1977), and show a faster response toward smiling faces and “likes”/thumbs up than adults (Demurie et al., 2012; Cromheeke and Mueller, 2016). Neuroimaging research has shown that adolescents, but not (young) adults, make more risky decisions in the presence of peers, which is supported by activation in reward-related neural circuitry (Chein et al., 2011). Together, these studies provide empirical evidence for an adolescent peak in sensitivity to a range of positive types of social rewards (i.e., likes, smiling faces, and potential approval from friends), yet few studies have examined age differences in the *subjective value* of social interactions (except Csikszentmihalyi et al., 1977).

Individual differences in sensitivity to social rewards have reliably been assessed using self-report in adolescents and adults with the Social Reward Questionnaire (SRQ; Foulkes et al., 2014b; SRQ-A; Foulkes et al., 2017). This questionnaire assesses five different types of social rewards, including the enjoyment of being flattered, liked, and gaining positive attention (*Admiration*), being cruel, callous, and using others for personal gains (*Negative Social Potency*), giving others control and allowing them to make decisions (*Passivity*), having kind, reciprocal relationships (*Prosocial Interactions*); and engaging in group interactions (*Sociability*). Thus, the SRQ assesses a broad set of social rewards that may underlie sensitivity to the social context. Prior work using the SRQ has shown meaningful differences in sensitivity to social rewards between adolescents with autism spectrum disorders and typically developing adolescents (i.e., enjoying passivity, but not engaging in group interactions; Van Hoorn et al., 2017) as well as a distinctive inverse pattern for adolescents high in callous-unemotional traits such that they enjoy being cruel, but not having kind relationships (Foulkes et al., 2017). To examine sensitivity to social rewards, the secondary aim of this paper was to validate our Dutch version of the SRQ-A and to examine test–retest reliability as well as construct validity using the Resistance to Peer Influence questionnaire (RPI; Steinberg and Monahan, 2007) and Behavior Inhibition Scale-Behavior Activation Scale (BIS-BAS; Carver and White, 1994) as a measure of sensitivity to non-social reward.

We expected a peak in sensitivity to all types of social rewards during adolescence, except for the rewarding feeling from giving others control (Passivity). For this more passive type of social reward, we expected a linear decrease given the importance of becoming independent from parents in adolescence into young adulthood (Crone and Dahl, 2012). In line with theory and empirical work, we expected that feeling rewarded when gaining positive attention (Admiration), enjoying kind relationships (Prosocial Interactions), as well as enjoying group interactions (Sociability) peak during adolescence and decrease again in young adulthood (Csikszentmihalyi et al., 1977; Chein et al., 2011; Demurie et al., 2012; Somerville, 2013; Cromheeke and Mueller, 2016). Finally, antisocial behaviors are also uniquely heightened during adolescence (Fairchild et al., 2013) and have been associated with feeling rewarded from cruel behavior toward others (Foulkes et al., 2014b; Craker and March, 2016). Therefore, we expected a peak in feeling rewarded from cruel behaviors toward others (Negative Social Potency) during adolescence.

With regards to gender, we expected specific differences in sensitivity to reward from prosocial behavior (Prosocial Interactions) and cruel behavior toward others (Negative Social Potency). Girls behave more prosocially across age and tend to be more supportive in their friendships compared to boys (Eisenberg et al., 1995, 2005; De Goede et al., 2009; Luengo Kanacri et al., 2013) whereas adolescent boys show more overt antisocial behavior compared to girls (Snyder et al., 2012). Thus, we expected that females would also be more sensitive to social rewards from prosocial interactions and that males would be more sensitive to rewards from cruel behaviors toward others.

## Materials and Methods

### Participants and Procedure

Participants were recruited from a large longitudinal brain imaging study with three time points called BrainTime. Recruitment for the BrainTime study occurred via high schools and advertisements in local newspapers in and around Leiden, the Netherlands. As part of the larger study, participants completed several online questionnaires, took part in a MRI study, and were compensated €10 per hour. Further recruitment details can be found in previous publications (e.g., Peters et al., 2016). The current cross-sectional study used the third time point of BrainTime, which consisted of 277 typically developing adolescents and young adults between 11 and 28 years old. Six participants from the BrainTime sample were excluded because of missing data for the SRQ-A. Hence, the final sample of the current study [called time point 1 (T1) for this paper] consisted of *N* = 271 participants [*M*_age_ = 17.84 years; *SD*_age_ = 3.67; range_age_ = 11.90–28.60 years; 144 females (53%)]. The sample consisted of 90% Caucasian participants, 6% non-Caucasian participants [Turkish (*n* = 1), Latin-American (*n* = 7), North-African (*n* = 1), African (*n* = 3), and Asian (*n* = 5)], and 4% of participants whose ethnicity was unknown. Participants in the sample had an average of 1.51 siblings (*SD* = 0.874, range = 0–5 siblings). There was no information about social economic status available for our participants.

A subset of 146 participants (52% of T1) also completed a follow up test–retest reliability session 6 months later, including several other questionnaires unrelated to this study (see e.g., Becht et al., 2018). Six participants were excluded because of incomplete data. Therefore, the final sample for the test–retest session [called time point 2 (T2) for this paper] included *N* = 140 participants [*M*_age_ = 18.48 years, *SD*_age_ = 4.07; range_age_ = 12.30–29.50 years; 79 females (56%)]. Of this sample, 94% of the participants were Caucasian, 6% of the participants was non-Caucasian [Latin-American (*n* = 2), North-African (*n* = 1), African (*n* = 2), and Asian (*n* = 3)], and the ethnicity of 1% of the participants was unknown.

To determine whether our sample was a normative Dutch sample, the intelligence of participants was estimated using subscales *Picture Completion* and *Vocabulary* of the WISC-III (11–16 year olds; Kort et al., 2002) or WAIS-III (16+ year olds; Uterwijk, 2000), at the second time point of the original BrainTime study. The estimated IQ scores fell within the average range (*N*_IQ_ = 239; *M*_IQ_ = 108.4; *SD*_IQ_ = 10.4). Prior to the study, all participants and/or parents of participants under 18 years old provided informed consent. For T1 of the current study, the Leiden University Medical Ethical Committee approved all procedures under the project name “Brain development between ages 8 and 25: A longitudinal study” with approval number P10.191. For the follow-up (T2), all procedures were approved by the Leiden University Ethical Committee under the name of “Braintime questionnaires” with approval number CEP16-0308/122.

### Questionnaire Development

#### Social Reward Questionnaire – Adolescent (SRQ-A) Version (Foulkes et al., 2017)

Participants aged 11–17 years completed the Dutch translation of the SRQ-A version (Foulkes et al., 2017) and participants aged 18+ years completed the Dutch translation of the adult SRQ (Brazil et al., in preparation). The two versions of the measure are highly similar (see the following paragraph). Similar to the original, the Dutch translation of the adult SRQ (Foulkes et al., 2014b; Brazil et al., in preparation) includes six subscales with a total of 23 questions: *Admiration* (enjoyment of being flattered, liked, and gaining positive attention, e.g., “I enjoy achieving recognition from others”); *Negative Social Potency* (enjoyment of being cruel, callous, and using others for personal gains, e.g., “I enjoy embarrassing others”); *Passivity* (enjoyment of giving others control over decisions, e.g., “I enjoy following someone else’s rules”); *Prosocial Interactions* (enjoyment of having kind, reciprocal relationships, e.g., “I enjoy treating others fairly”); *Sexual Relationships* (enjoyment of having frequent sexual experiences, e.g., “I enjoy having an active sex life”); and *Sociability* (enjoyment of engaging in group interactions, e.g., “I enjoy going to parties”).

The Dutch translation of the adolescent SRQ (SRQ-A) was translated by a bilingual Dutch-English speaker using the forward–backwards method (Bracken and Barona, 1991). The last author checked with Foulkes and Brazil to make sure that the translated items reflected the content of the original items, and that the adolescent and adult version used similar wording. In line with the English SRQ-A, the *Sexual Relationship* subscale was removed, and minor word changes were made to improve clarity for younger participants. Care was taken that all participants understood the instructions of the questionnaire. Responses to the adult and adolescent questionnaires were coded on a seven-point Likert scale, ranging from 1 = strongly disagree to 7 = strongly agree. Mean scores for each subscale are calculated, but no overall mean score is computed due to the contrasting meaning of some of the subscales (cf. Foulkes et al., 2014b, 2017).

### Measures to Assess Construct Validity of Dutch SRQ-A

#### Resistance to Peer Influence (RPI; Steinberg and Monahan, 2007)

This questionnaire provided a general measure of resistance to peer influence (RPI). In 10 pairs of statements, participants indicated which of the two statements applied to them the most, e.g., “Some people go along with friends just to keep their friends happy” but “Other people refuse to go along with what their friends want to do, even though they know it will make their friends unhappy.” After selecting a statement, participants decided whether it was “really true” or “sort of true” for them. Afterward, responses were coded on a four-point scale and averaged, with a high RPI score indicating high RPI. Prior research shows that adolescents with lower scores on the RPI (more susceptible to peer influences) are more impulsive and take more risks (Steinberg and Monahan, 2007). Therefore, we expected that adolescents who are more resistant to peer influence (high RPI scores) would have higher Prosocial Interactions scores and lower Sociability scores, since they may place more value on the opinions of others and use these opinions to guide their behavior.

#### Behavioral Inhibition System–Behavioral Activation System (BIS–BAS; Carver and White, 1994)

This is a 24-item questionnaire that measures both the Behavioral Inhibition System (BIS) and Behavioral Activation System (BAS). It consists of four subscales; *BIS* (reactions to the anticipation of punishment), *BAS Drive* (the persistent pursuit of desired goals), *BAS Fun Seeking* (desire for new rewards and willingness to approach a potentially rewarding event), and *BAS Reward Responsiveness* (sensitivity to pleasant reinforcers in the environment). Items consist of several statements and participants had to indicate to what extent they agreed with each statement on a four-point scale (1 = strongly agree, 4 = strongly disagree). We expected that BAS Reward Responsiveness would only be related to more positive types of social reward, including feelings of reward from getting positive attention (Admiration), Prosocial Interactions, and engaging in group interactions (Sociability). Moreover, we expected that BAS Drive and BAS Fun Seeking would be related to all SRQ-A subscales, because they measure trait-like sensitivity to rewards, which may underlie sensitivity to social rewards. We did not expect any relationships between BIS and social rewards.

### Statistical Analyses

#### Validity and Reliability of SRQ-A

To validate the Dutch SRQ-A for both adolescents and young adults, we used R studio with the Lavaan package to run a confirmatory factor analysis (CFA; Rosseel, 2012). At T1 (*N* = 271), 157 adolescents completed the 20-item SRQ-Adolescent and 114 adults completed the 23-item adult SRQ. Given that the “Sexual Relationships” scale is only included in the adult version, these questions were excluded from current analyses. Therefore, our model consisted of 50 parameters (i.e., 20 factor loadings, 20 error variances, 10 factor correlations). Given that the subjects-to-parameters ratio should be at least 5:1 (Bentler and Chou, 1987) our sample was adequate to test this model (ratio 5.4:1). The SRQ-A consists of ordinal items and therefore the mean and variance adjusted weighted least squares (WLSMV) estimation procedure was used (Flora and Curran, 2004). A comparative fit index (CFI) of 0.95 or higher and a root mean square error of approximation (RMSEA) of 0.08 or lower were used to determine a good model fit (Hu and Bentler, 1999), as in the original validation papers.

Internal consistency was assessed using Cronbach’s alpha. However, given the limitation that Cronbach’s alpha is not an indicator of scale unidimensionality (Schmitt, 1996), we relied most on mean inter-item correlations (MICs) to assess homogeneity and internal consistency of the scales (cf. Foulkes et al., 2017). For the sake of completeness, we also report Cronbach’s alphas and MICs split for age groups in Supplementary Table 1. Construct validity was tested with the additional questionnaires (RPI and BIS-BAS) completed by all participants at T1, using Pearson’s correlations in IBM SPSS Statistics 23. Test–retest reliability was assessed by correlating the subscale scores of the follow-up session at T2 with the subscale scores of the initial session for each participant. To control for errors resulting from multiplicity, the false discovery rate (FDR) was used (Benjamini and Hochberg, 1995).

#### Age and Gender Effects

We expected nonlinear age effects for all types of social reward assessed with the SRQ-A, except Passivity for which we expected a linear decrease with age. Therefore, we used a regression analysis with the enter method in SPSS for each subscale separately, and included effects of gender in model 1, adding linear and quadratic age effects in model 2, and finally the interaction effects of gender × linear age, and gender × quadratic age in model 3.^1^ The social reward subscales were utilized as the dependent variable, and age, gender, and the interaction terms of age × gender were added as independent variables. Age was centered because we included interaction terms in our models (Aiken and West, 1991).

## Results

### Validation of Dutch SRQ-A

In order to ensure that the Dutch version of the SRQ-A was a valid and reliable measure of social rewards we tested a five-factor model using a CFA, based on the five-factor model of the original SRQ-A. The items and factors used in the CFA corresponded with the original SRQ-A. The CFA-model fit the data well [$\chi_{\left(160\right)}^{2}$ = 375.05, *p* < 0.001; CFI = 0.96; RMSEA = 0.065, 90% CI = 0.067-0.087]. The ranges of the factor loadings were between 0.44 and 0.90 (*M*_loadings_ = 0.67, *SD*_loadings_ = 0.11). All factor loadings are shown in Table 1.

**TABLE 1:** Standardized factor loadings from the five-factor CFA.

**Factor**	**Loading**	**Item number**
Prosocial interaction	0.65	2
	0.65	6
	0.54	16
	0.65	19
	0.68	22
Passivity	0.85	12
	0.76	21
	0.72	23
Admiration	0.66	1
	0.69	7
	0.73	11
	0.62	18
Sociability	0.61	4
	0.58	10
	0.90	15
Negative social potency	0.70	3
	0.44	5
	0.77	8
	0.47	14
	0.62	17

*Item numbers are based on the adult SRQ. Items 9, 13, and 20 correspond with the sexual relationships subscale and are not included.*

#### SRQ-A Reliability

In Tables 2, 3, an overview of correlations, descriptive statistics, Cronbach’s alphas, and MICs for each of the five subscales is displayed. At T1, internal consistency of four out of five subscales was reasonable, with Cronbach’s alphas between 0.67 to 0.78 (Taber, 2018), and Negative Social Potency had a slightly lower alpha (α = 0.55, *SD* = 0.07). At T2, internal consistency for all five subscales was reasonable, with Cronbach’s alphas between 0.67 and 0.84. The MICs fell in the acceptable range for all subscales for T1 and T2 (T1: range = 0.21-0.55; T2: range = 0.33–0.49) conform guidelines from Clark and Watson (1995) for subscales that measure relatively narrow constructs.

**TABLE 2:** Correlations of each subscale at T1 (*n* = 271), and Pearson’s correlations between mean subscale scores at T1 and T2 (*n* = 140).

	**1**	**2**	**3**	**4**	**T1−T2**
1. Admiration					0.63^∗∗∗^
2. Negative social potency	**0.18^∗∗^**				0.69^∗∗∗^
3. Passivity	−0.03	−0.08			0.56^∗∗∗^
4. Prosocial interactions	**0.40^∗∗^**	**−0.19^∗∗^**	<0.01		0.58^∗∗∗^
5. Sociability	**0.47^∗∗^**	0.07	−0.02	**0.28^∗∗^**	0.65^∗∗∗^

*Factor correlations with *p* < 0.05 are shown in bold. ^∗∗^*p* < 0.01, ^∗∗∗^*p* < 0.001.*

**TABLE 3:** Descriptive statistics (minimum, maximum, mean, and SD), mean inter-item correlations (MICs), and Cronbach’s alphas of each subscale at T1, as well as MICs and Cronbach’s alphas at T2.

	**Minimum T1**	**Maximum T1**	**Mean^+^ (*SD*) T1**	**MIC T1**	**MIC T2**	**Cronbach’s alpha T1**	**Cronbach’s alpha T2**
**Social Reward Questionnaire – Adolescents (SRQ-A)**							
Admiration	1.25	7.00	5.18 (*1.04*)	0.34	0.41	0.69	0.73
Negative social potency	1.00	4.80	2.08 (*0.77*)	0.21	0.33	0.55	0.67
Passivity	1.00	6.00	2.84 (*1.17*)	0.55	0.63	0.78	0.84
Prosocial interactions	3.00	7.00	6.04 (*0.68*)	0.31	0.38	0.67	0.74
Sociability	1.00	7.00	5.61 (*1.07*)	0.41	0.49	0.68	0.74

*+, Mean item score in each factor.*

#### SRQ-A Test–Retest Reliability

Test–retest reliability was assessed with Pearson correlations (cf. Foulkes et al., 2017) based on 140 participants who completed the SRQ-A again roughly 6 months after the initial assessment (*M*_T1–T2_ = 6.96 months, *SD*_T1–T2_ = 1.92 months, range = 3.36–12.00 months). Pearson correlations were in the moderate range (Mukaka, 2012) for each subscale (*M* = 0.62, *SD* = 0.05, all *ps* < 0.001), which indicates that the questionnaire is relatively stable across 6-months’ time (Table 2).

#### SRQ-A Construct Validity

To examine the associations between social rewards and sensitivity to social context and non-social reward, we conducted Pearson correlation analyses. FDR-corrected *p*-values are presented in Table 4. Both *Admiration* and *Sociability* were positively correlated with all BAS subscales. *Sociability* was also negatively correlated with RPI. *Negative Social Potency* was positively correlated with BAS Drive and BAS Fun Seeking. *Passivity* was negatively correlated with BAS Drive and BAS Fun Seeking. Finally, *Prosocial Interactions* was positively correlated with all measures. Findings were in the expected direction and imply an acceptable construct validity of the Dutch SRQ-A.

**TABLE 4:** Pearson correlations between SRQ-A subscales and external measures.

	**SRQ-A subscale**				
	**Admiration**	**Negative social potency**	**Passivity**	**Prosocial interactions**	**Sociability**
**RPI**					
Mean RPI	–0.01	–0.08	–0.08	**0.22^∗∗^**	**−0.24^∗∗^**
**BISBAS**					
BAS drive	**0.38^∗∗^**	**0.19^∗∗^**	**−0.25^∗∗^**	**0.23^∗∗^**	**0.15^∗∗^**
BAS fun seeking	**0.35^∗∗^**	**0.17^∗∗^**	**−0.18^∗∗^**	**0.24^∗∗^**	**0.26^∗∗^**
BAS reward responsiveness	**0.41^∗∗^**	0.00	–0.04	**0.35^∗∗^**	**0.32^∗∗^**
BIS	0.09	–0.11	0.10	**0.21^∗∗^**	0.05

Significant correlations after FDR correction for multiple comparisons (with alpha level 0.05) in in bold. ^*^p < 0.05; ^∗∗^*p* < 0.01.

### Age and Gender Effects in Sensitivity to Social Reward

To examine age and gender effects on sensitivity to social reward, separate regression analyses were conducted for each SRQ-A subscale. Analyses included gender in model 1 as a baseline, linear and quadratic age effects in model 2, and interaction effects of gender × linear age and gender × quadratic age in model 3 (see Table 5 for an overview of all models per subscale).

**TABLE 5:** Regression analysis (enter method) per subscale separately.

	**SRQ-A subscale**														
	**Admiration**			**Negative social potency**			**Passivity**			**Prosocial interactions**			**Sociability**		
	***B***	***SE.B***	**β**	***B***	***SE*.*B***	**β**	***B***	***SE*.*BB***	**β**	***B***	***SE*.*B***	**β**	***B***	***SE*.*B***	**β**
**Model 1**															
Constant	5.20	0.09		1.91	0.06		2.80	0.10		6.24	0.05		5.70	0.09	
Gender	−0.04	0.13	−0.02	0.36	0.09	0.23^∗∗^	0.09	0.14	0.04	−0.43	0.08	−0.32^∗∗^	−0.20	0.13	−0.09

*$R_{a\,d\,j}^{2}$*		−0.00			0.05			−0.00			0.10			0.01	

**Model 2**															
Constant	**5.32**	**0.10**		1.96	0.07		**2.70**	**0.11**		**6.26**	**0.06**		5.73	0.10	
Gender	**−0.05**	**0.12**	**−0.03**	0.37	0.09	0.24^∗∗^	**0.07**	**0.14**	**0.03**	**−0.44**	**0.08**	**−0.32^∗∗^**	**−**0.20	0.13	**−**0.09
Age (linear)	**0.07**	**0.02**	**0.25^∗∗^**	**−**0.02	0.01	**−**0.11	**0.04**	**0.02**	**0.12**	**0.04**	**0.01**	**0.23^∗∗^**	**−**0.01	0.02	**−**0.03
Age (quadratic)	**−0.01**	**0.00**	−0.16^*^	**−**0.00	0.00	**−**0.09	**0.01**	**0.00**	0.14^*^	**−0.00**	**0.00**	**−0.04**	**−**0.00	0.00	**−**0.04

*$R_{a\,d\,j}^{2}$*		**0.04**			0.07			**0.04**			**0.14**			0.00	

**Model 3**															
Constant	5.33	0.11		**1.95**	**0.08**		2.66	0.13		6.29	0.07		5.74	0.12	
Gender	−0.07	0.16	−0.03	**0.41**	**0.11**	**0.27^∗∗^**	0.11	0.18	0.05	−0.49	0.10	−0.36^∗∗^	−0.19	0.17	−0.09
Age (linear)	0.06	0.03	0.20^*^	−**0.06**	**0.02**	−**0.27^∗∗^**	0.06	0.03	0.19	0.04	0.02	0.22^*^	−0.02	0.03	−0.08
Age (quadratic)	−0.01	0.01	−0.19	−**0.00**	**0.00**	−**0.09**	0.01	0.01	0.20	−0.00	0.00	−0.11	−0.00	0.01	−0.05
Gender × age (linear)	0.03	0.04	0.07	**0.08**	**0.03**	**0.27^∗∗^**	−0.05	0.04	−0.11	0.00	0.02	0.01	0.03	0.04	0.08
Gender × age (quadratic)	0.00	0.01	0.02	**−0.00**	**0.01**	**−0.07**	−0.00	0.01	−0.05	0.00	0.01	0.10	0.00	0.01	−0.00

*$R_{a\,d\,j}^{2}$*		0.03			**0.10**			0.04			0.13			0.00	

Best-fitted models are displayed in bold. No effects are found for sociability, and therefore, no model is displayed in bold. Age is centered (mean = 17.84 years old). Gender is coded 0 = female, 1 = male. ^*^p < 0.05; *^∗∗^p* < 0.01.

**FIGURE 1:**
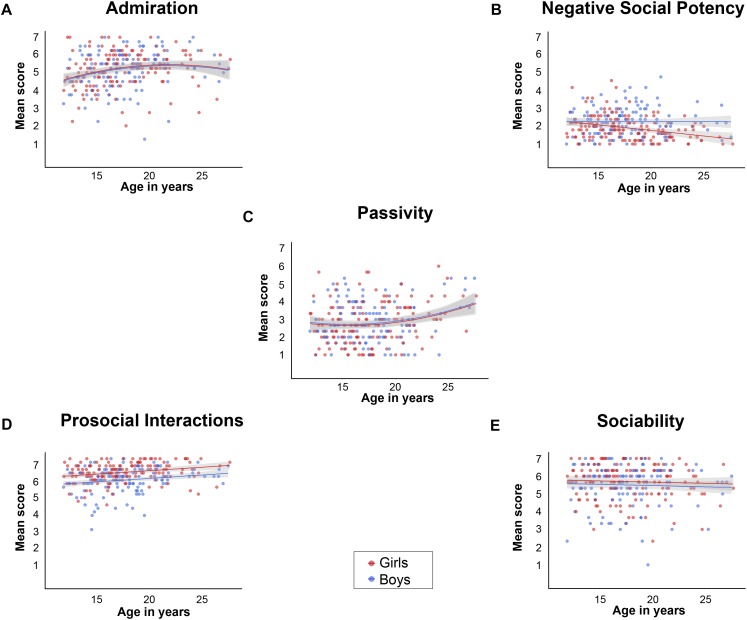
Mean scores on each SRQ-A subscale of adolescents and young adults between ages 11–28 years. **(A)** Mean scores of *Admiration* showing a quadratic age effect, with a peak at 21.34 years old. **(B)** Mean scores of *Negative Social Potency* showing an interaction effect of gender and age. **(C)** Mean scores of *Passivity* showing a quadratic age effect, with a dip at 15.40 years old. **(D)** Mean scores of *Prosocial Interactions* showing a main effect for gender and a main effect of age, and **(E)** mean scores of *Sociability* showing no main nor interaction effects.

For *Admiration*, the second and third model were significant (*p* < 0.01 and *p* = 0.02, respectively), but only the second model predicted significantly more variance than the baseline model [*F*(3,267) = 4.49, *p* < 0.01, *$R_{\mathrm{adj}}^{2}$* = 0.04, *$R_{\mathrm{change}}^{2}$* = 0.05], hence we picked the most parsimonious model. The results showed a quadratic age effect (β = −0.16, *t* = −2.36, *p* = 0.02), indicating an adolescent peak in late adolescence which fell at 21.34 years old (Figure 1A). This suggests that the enjoyment of *Admiration* increases for both boys and girls until young adulthood, and levels off after the age of approximately 21.34 years old.

The regression analysis for *Negative Social Potency* resulted in three significant models (all *p* < 0.001). The third model explained significantly more variance than the baseline model [*F*(5,265) = 6.77, *p* < 0.01, *$R_{\mathrm{adj}}^{2}$* = 0.10, *$R_{\mathrm{change}}^{2}$* = 0.03], with main effects of age (β = −0.27, *t* = −3.11, *p* < 0.01) and gender (β = 0.27, *t* = 3.60, *p* < 0.01) which were qualified by an interaction of linear age × gender (β = 0.27, *t* = 2.88, *p* < 0.01). The interaction revealed that boys and girls show similar levels of *Negative Social Potency* in early adolescence, with patterns diverging later in adolescence when girls show a decrease, while boys show no changes over time (Figure 1B).

For *Passivity*, all three models were significant, with the second model predicting significantly more variance than the baseline model [*F*(3,267) = 4.99, *p* < 0.01, *$R_{\mathrm{adj}}^{2}$* = 0.04, *$R_{\mathrm{change}}^{2}$* = 0.05]. The results showed a quadratic effect of age (β = 0.14, *t* = 2.05, *p* = 0.04), revealing an adolescent dip in mid-adolescence at 15.40 years old (Figure 1C). This suggests that the enjoyment of *Passivity* decreases until approximately age 15.40 years, and increases again with age, for both boys and girls.

The regression analysis for *Prosocial Interactions* resulted in three significant models, with the second model explaining significantly more variance [*F*(3,267) = 15.06, *p* < 0.01, *$R_{\mathrm{adj}}^{2}$* = 0.14, *$R_{\mathrm{change}}^{2}$* = 0.05], by a main effect of linear age (β = 0.23, *t* = 3.54, *p* < 0.01) and gender (β = −0.32, *t* = −5.70, *p* < 0.01). These findings show that girls enjoy *Prosocial Interactions* more across all ages, and in addition, that both boys and girls have higher levels of *Prosocial Interactions* with age (Figure 1D).

Finally, the regression analysis for *Sociability* revealed no significant model, indicating neither significant main effects nor interaction effects of age and gender (all *ps* > 0.13). This suggests that enjoyment of engaging in group interactions is stable across adolescence and into young adulthood (Figure 1E).

## Discussion

The main goal of the present study was to examine age and gender differences in sensitivity to different types of social rewards in a sample of adolescents and young adults between the ages of 11 and 28 years. Understanding sensitivity to social reward as an underlying neurocognitive mechanism for social influence processes is vital to further delineate why and under what conditions adolescents are affected by their social context (Somerville et al., 2018). Our key finding is that the reward from being liked and gaining positive attention showed a late adolescent peak. Gender differences were in the expected direction, as girls felt more rewarded by kind interactions and this increased with age, whereas enjoying being cruel to others was stable for boys and decreased for girls with age. However, contrary to our expectations, social reward from engaging in group interactions was stable across the entire age range, and letting others make decisions showed a mid-adolescent dip. Thus, sensitivity to social reward is a nuanced and complex phenomenon, which reveals differential age-related patterns for each type of social reward. These findings are further unpacked below.

### Social Reward as an Underlying Neurocognitive Mechanism for Social Influence Processes

The present study was the first to study the *subjective value* of a broad range of social rewards in a cross-sectional sample that spanned early adolescence to adulthood. Our findings revealed that the reward from being liked and gaining positive attention showed a higher hedonic value during late adolescence (at approximately age 21 years). Given that previous work provides empirical evidence for an early to mid-adolescent peak in neural reward sensitivity (approximately age 16–17 years; e.g., Braams et al., 2015; Silverman et al., 2015), peer influence on risk perception and prosocial behavior (age 12–14 years; Knoll et al., 2015; age 12–13 years; Van Hoorn et al., 2016a) as well as sensitivity to peer influence (age 10–14 years; Steinberg and Monahan, 2007), this peak fell somewhat later than expected.

Sensitivity to social evaluation is thought to be central throughout adolescence (Somerville et al., 2013), but younger adolescents are found to be most sensitive to social exclusion (Sebastian et al., 2010). As such, social signals of positive attention may be particularly important during early adolescence because this is a period of rapid social development, without necessarily increasing in hedonic value (Foulkes and Blakemore, 2016). Possibly, early adolescents’ sensitivity to social influences are guided by greater motivations to avoid social punishment or risk (i.e., social exclusion), rather than an orientation to social reward (Blakemore, 2018). Speculatively, the “balance” between *avoiding* social risk and *gaining* social approval as processes that predict sensitivity to the social context changes with age. The increase in hedonic value of social approval during late adolescence fits with the epidemiological literature on morbidity and mortality from risk taking which peaks in late adolescence (Willoughby et al., 2013). Together, this work illustrates that the emergence of reward-related behaviors such as risk taking likely depends on age, and also on opportunities and characteristics of the social context (Willoughby et al., 2013).

Next, our findings revealed that early adolescents *and* young adults felt more rewarded when giving others control over decisions (i.e., passive behavior), compared to mid-adolescents (approximately age 15 years). While the decrease during adolescence corroborates previous research emphasizing that adolescents seek independence and strive to become more autonomous (Zimmer-Gembeck and Collins, 2003), it was somewhat surprising that our findings revealed an adolescent dip rather than a linear decrease with age. Interestingly, Foulkes et al. (2017) noticed a similar pattern in the relationship between psychopathic traits and passivity, which were positively related in adults, but negatively related in adolescents. Young adults tend to have control over most of their life decisions, possibly resulting in more enjoyment when giving others control over decisions, as this means less effort for the individual. However, passivity in adolescents may be experienced as submission to authority figures such as parents, which is undesirable in the context of establishing their independence (Foulkes et al., 2017).

Moreover, late adolescents and young adults experienced being in positive, reciprocal relationships as more rewarding compared to younger adolescents. Gradual improvement in mentalizing skills across adolescence into young adulthood may facilitate positive interactions with others (Frith and Frith, 2006), and these positive experiences may in turn feel rewarding. These findings are partly consistent with prior research showing that prosocial behavior (i.e., behavior that benefits others) increases during young adulthood after a dip during adolescence (although note that prosocial *behavior* is different from *enjoying* prosocial relations; Eisenberg et al., 2005; Luengo Kanacri et al., 2013). Hence, prosocial behavior observed in late adolescence and adulthood may perhaps in part be driven by experiencing more reward from this behavior than younger adolescents. In line with our expectations, we found gender differences in social reward from experiencing kind relationships as well as being cruel toward others. Across adolescence and young adulthood, girls feel more rewarded from having intimate, reciprocal interactions than boys. This resonates with previous work indicating that girls behave more prosocially and show more intimacy and support in their friendships (Eisenberg et al., 1995; De Goede et al., 2009).

Further, we observed that the rewarding feeling from engaging in group interactions does not show age-related changes in hedonic value. Previous studies have shown that different social actors within the social context have different effects on adolescent decision-making (van Hoorn et al., 2019). For example, peers can create vulnerabilities and opportunities for adolescents (Van Hoorn et al., 2016b), and the presence of a mother or other adult differentially modulates reward-related neural circuits in the brain than peers (Chein et al., 2011; Guassi Moreira and Telzer, 2016; van Hoorn et al., 2018). The SRQ-A does not distinguish between reward value from interacting with peers, strangers, and parents, as it measures reward value from social interactions in general. This likely contributed to the differences in the current findings relative to work from Csikszentmihalyi et al. (1977), who reported increased reward in adolescence specifically during conversations with peers relative to adults.

Finally, we examined one relatively negative type of social reward, i.e., feeling reward from being cruel to others. Both males and females in our typically developing sample reported a limited sense of reward when being cruel, callous, and using others for personal gains, which decreased with age for females while it was stable for males. Although adolescence is a time during which antisocial behavior peaks (Fairchild et al., 2013), the current findings do not provide evidence for a heightened feeling of reward from being cruel and using others for personal gains during this period. As such, the increase in antisocial behavior during adolescence may not due to more enjoyment of behaving antisocially, at least not in a normative sample, highlighting the importance of social context in which these types of behavior occur.

### Validation of the Dutch SRQ-A and Relation With Non-social Reward

Our analyses indicated that the Dutch translation of the SRQ-A is a valid and reliable measure of sensitivity to social reward in adolescence. We further examined the relationship between social rewards and RPI (Steinberg and Monahan, 2007) as well as non-social rewards (BIS-BAS; Carver and White, 1994). RPI was associated with two types of social rewards that are most directly related to friendships and being part of a group. Feeling more rewarded from engaging in group interactions was associated with less RPI, which likely reflects a higher tendency to conform to the peer group if an adolescent highly values the (opinions from) the peer group (Telzer et al., 2018). On the other hand, feeling more rewarded from prosocial interactions was related to greater RPI. Speculatively, adolescents who enjoy prosocial and kind interactions potentially have more of these positive friendships, which are known to provide a buffer against negative behaviors such as risk taking (Telzer et al., 2015).

In terms of non-social reward, sensitivity to pleasant reinforcers in the environment (BAS Reward Responsiveness) was only related to more positive types of social reward, including feelings of reward from getting positive attention, prosocial interactions, and engaging in group interactions. Across the entire range of social rewards that we measured, each subtype was related to the drive or persistent pursuit of seeking out rewards (BAS Drive) and the motivation to find novel rewards spontaneously (BAS Fun Seeking). This is in line with our expectations, and serves to support the idea that the SRQ-A measures reward value. The underlying construct for sensitivity to social reward may be the tendency to seek out rewards, both in more spontaneous and persistent ways (Carver and White, 1994), rather than the avoidance of punishment (BIS), which did not show this consistent (reverse) association with social rewards. Taken together, the relations between social reward and non-social reward as well as RPI are in the expected direction and provide interesting avenues for future research.

### Limitations and Future Directions

It is important to acknowledge the limitations of our study. Sensitivity to social reward may be affected by earlier experiences, such as early stressful life events (see e.g., Coker et al., 2011). While this was beyond the scope of the current paper, it would be an interesting future direction. Moreover, the SRQ-A does not distinguish between reward value from interacting with different actors such as peers and parents, as it was designed to measure reward value from social interactions in general. A promising avenue for future research is to examine social reward from specific others (peers, parents, strangers, best friends, etc.) in a wide adolescent age range (also see Güroğlu et al., 2014). These results will be important to better understand adolescent-specific behavior for each type of social rewards within different social contexts. Finally, our results are based on cross-sectional data and did not include a younger comparison group of children younger than age 11 years. Given potential issues associated with lower internal consistency in younger adolescents, it will be important to develop additional items that are suitable for children and young adolescents. To further understand the developmental pattern of the different social rewards, future studies should employ a longitudinal design with children, adolescents, and adults.

## Conclusion

Theoretical and empirical work characterizes adolescence as a time of uniquely heightened sensitivity to (non-social) reward, social stimuli, and peer influence (Galvan, 2010; Chein et al., 2011; Blakemore and Mills, 2014). The present study was the first to examine subjective sensitivity to social rewards in a cross-sectional sample between early adolescence and adulthood. Our findings revealed that reward from being liked and gaining positive attention showed a higher hedonic value during late adolescence, which corroborates the idea that sensitivity to the social context may at least partly due to the social reward of getting approval from others. However, at the same time the results highlight that social reward is more nuanced and complex (cf. Foulkes and Blakemore, 2016), because this pattern was not apparent in other types of social rewards that were examined. The SRQ-A provides an important individual differences measure in typically developing samples as well as atypical samples where social reward may go awry, such as autism spectrum disorders.

## Data Availability

The datasets generated for this study are available on request to the corresponding author.

## Ethics Statement

Prior to the study, all participants and/or parents of participants under 18 years old provided written informed consent in accordance with the Declaration of Helsinki. For the first time point (T1) of the current study, the Leiden University Medical Ethical Committee approved all procedures under the project name “Brain development between ages 8 and 25: A longitudinal study” with approval number P10.191. For the follow-up (T2), all procedures were approved by the Leiden University Ethical Committee under the name of “Braintime questionnaires” with approval number CEP16-0308/122.

## Author Contributions

JvH and EC contributed to the conception and design of the study with input from LF on questionnaire design. SA and JvH performed the statistical analyses with input from MB. SA and JvH wrote the manuscript. LF, MB, and EC provided important intellectual content to the manuscript. All authors contributed to the manuscript revision, and read and approved the submitted version of the manuscript.

## Conflict of Interest Statement

The authors declare that the research was conducted in the absence of any commercial or financial relationships that could be construed as a potential conflict of interest. The handling Editor declared a past co-authorship with one of the authors EC.

## Footnotes

1 We also ran regression models controlling for self-reported psychopathology (*N* = 18; coded as 0 = no psychopathology; 1 = psychopathology). These analyses yielded the same results with age and gender as those without psychopathology. For the subscale Sociability, we found a small main effect of psychopathology (β = −1.84, *$R_{\mathrm{adj}}^{2}$* = 0.03, *p* = 0.002).
