# Supplementary material for: Age and Gender Effects in Sensitivity to Social Rewards in Adolescents and Young Adults
Source: Front Behav Neurosci. 2019 Jul 29;13:171. doi: 10.3389/fnbeh.2019.00171 (PMC6681770; doi:10.3389/fnbeh.2019.00171)
Supplement: Supplementary file 1 [file Table_1.DOCX]

**Age and Gender Effects in Sensitivity to Social Rewards in Adolescents and Young Adults**

**Supplementary materials**

*Supplementary Table 1.* Cronbach’s alphas and Mean Inter-Item Correlations for each subscale split per age group.

| A \| Cronbach’s Alphas | | | | |
| --- | --- | --- | --- | --- |
| Subscale | Age group 11-14  (*N* = 66) | Age group 15-17  (*N* = 90) | Age group 18-22  (*N* = 87) | Age group 23+  (*N* = 28) |
| Admiration | .49 | .74 | .80 | .70 |
| Negative Social Potency | .45 | .57 | .67 | .41 |
| Passivity | .72 | .84 | .68 | .87 |
| Prosocial Interactions | .71 | .59 | .70 | .72 |
| Sociability | .61 | .63 | .78 | .56 |
| B \| Mean Inter Item Correlations (MIC) | | | | |
| Admiration | .19 | .42 | .49 | .36 |
| Negative Social Potency | .16 | .20 | .34 | .16 |
| Passivity | .46 | .63 | .42 | .70 |
| Prosocial Interactions | .34 | .26 | .34 | .32 |
| Sociability | .35 | .36 | .54 | .30 |
